# The growth of recovery capital in clients of recovery residences in Florida, USA: a quantitative pilot study of changes in REC-CAP profile scores

**DOI:** 10.1186/s13011-022-00488-w

**Published:** 2022-08-06

**Authors:** Sofia Härd, David Best, Arun Sondhi, John Lehman, Richard Riccardi

**Affiliations:** 1 Department of Social Work, Stockholm University, SE-106 91 Stockholm, Sweden; 2 Department of Criminology, Investigation and Policing, Room 16, Trinity Building, Leeds Trinity University, Horsforth, Leeds, LS18 5HD UK; 3 Therapeutic Solutions (Addictions), 4 Old Park Lane, London, W1K 1QW UK; 4 Recovery Outcomes Institute (ROI), Inc, Boynton Beach, Florida USA; 5 Fellowship Recovery Community Organization, Inc, Margate, Florida USA

**Keywords:** REC-CAP, Recovery residence, Recovery capital, Retention

## Abstract

**Background:**

There is a growing evidence base around predictors of retention and completion in a range of recovery residence models, particularly Oxford Houses and Sober Living Houses, and recovery housing is recognized as a clearly evidenced area of recovery intervention. The aim of the study was to quantitatively assess recovery capital in a sample of recovery residence clients.

**Method:**

The study used a repeated measures self-completion of a standardized recovery capital instrument (REC-CAP) for clients retained across various houses within one Level 2 recovery residence provider whose program was based on a 12-step approach. While 823 clients participated in the baseline assessment, a sample of 267 clients was achieved for six-month follow-up interview, based on those retained in the residence. A logistic regression model examined factors associated with retention and a repeated measures marginal mixed model evaluated the factors associated with changes in recovery capital between the baseline and the follow-up assessment.

**Results:**

Members of the group that remained in recovery residences were more likely to be older with a record of high participation in recovery groups, with greater drop-out among younger residents, female residents and those with an identified housing need. For those retained to follow-up, greater recovery capital growth was associated with employment, higher levels of social support and more recovery group involvement, as well as age and a higher quality of life. The need for family support was shown to reduce levels of recovery capital. However, those younger people who were retained reported better recovery capital growth during the initial six months of residence.

**Conclusion:**

The key conclusion is that while recovery capital generally increases during a stay in a recovery residence, it does not do so consistently across the sample population. This has implications for how pathways to recovery group engagement are supported for women and young people and how social support (encompassing housing, employment and family issues) is provided to those populations during periods of residence. This suggests the potential need for training and guidance for house managers working with these groups.

## Introduction

Recovery, as a social movement for change [1], has gained increased prominence following a re-evaluation of the meaning of recovery and further questioning of the methods used to measure recovery progress, based, as recovery residences exemplify, on the centrality of peer processes [2]. This prominence derives from a growing evidence base of peer-supported recovery initiatives, including recovery residences [3] and the emerging evidence around recovery community organisations [4]. This development can be described as a transition from a deficit to a strengths-based model predicated on the idea that recovery is a process that takes time (according to the Betty Ford Consensus Panel [5] the typical duration is around five years, with significantly reduced relapse risk beyond this point), and happens between people and within communities [6]. Braithwaite [7] has argued that the transitions seen in restorative justice, therapeutic jurisprudence, positive criminology and both mental health and addiction recovery are parts of a paradigmatic transition to a community-focused model of non-domination that is predicated on social and structural solutions that are empowering and inclusive. However, it is important to note that, as Davidson et al. [8] have pointed out, there have been considerable criticisms of the recovery movement as something that has been manipulated politically, with the ‘responsibility’ component of recovery used as a justification for reductions in funding and service provision and the recovery concept being linked to a neo-liberal political agenda.

One illustration of the theoretical developments regarding recovery is the concept of recovery capital. The origins of this concept derive largely from two core concepts of social capital. Bourdieu [9] argued that a sense of belonging, trust and tolerance are key resources particularly in communities where there is a lack of financial capital and limited access to resources. For Putnam [10], social capital was both a resource that individuals can draw upon but also as a commitment to the group and reciprocity is an essential feature of social capital. Putnam [11] differentiated between ‘bonding’ capital (the strength of links within established groups) and ‘bridging’ capital which refers to the links and associations between groups. Putnam’s work suggests that individuals from marginalised communities can have strong bonds but still have little access to community resources if there are not bridges to more connected and engaged groups.

This is reflected in the concept of recovery capital defined as the different types of resources an individual can draw upon to initiate and sustain recovery [12]. Cloud and Granfield [12] divide an individual’s resources into four categories: social, human, cultural and physical capital. These categorizations have developed since the concept was first introduced, and different categorizations have been used to classify the resources. White & Cloud [13], for example, suggest social, personal and community capital. However, the Cloud and Granfield paper [12] also introduced the concept of ‘negative recovery capital’ to refer to barriers to recovery with the authors providing four examples – older age, female gender, a history of incarceration and significant mental health problems. While the concept of negative recovery capital has raised questions about a ‘strengths-based’ recovery model, Fomiatti, Moore and Fraser [14] have gone further in suggesting that the measurement of recovery capital ignores the structural predictors of wellbeing including deprivation and inequality and over-emphasises personal choice in recovery pathways.

Notwithstanding this ongoing debate, a number of assessment tools have been developed to assess recovery capital taking a strength-based approach [15 16 17 18 19]. Hennessy [20] conducted a systematic review of the literature on recovery capital, focused primarily on the principles of recovery capital as a theoretical framework. This overview suggests that multiple recovery capital models are being used involving a variety of resources from the individual, micro- and meso-levels, although individual-level resources are the most prevalent. Developments can also be seen in the interpretation of different resources, which now include both general recovery-related factors, as originally outlined by Cloud and Granfield [12], and more narrow, treatment-specific interpretations [21].

Hennessy suggests that recovery capital is dynamic, in that it can either increase or decrease over time, and that factors in recovery capital tend to interact with each other, and with other external factors. Witbrodt et al. [22] examine the effectiveness of Motivational Interviewing Case Management (MICM), and whether this varies according to levels of recovery capital. Clients with high levels of recovery capital were found to be likely to improve as a result of participation in MICM, whereas clients with low recovery capital performed no better than other, more common treatments.

Recovery capital is likely to vary at different stages of the recovery change process [23, 24] and across different sub-populations. Growth in recovery capital should therefore be viewed as an ongoing process rather than a stable state, and should be considered bi-directional between recovery capital and other markers of wellbeing [20]. Other factors shown to influence the level of recovery capital and change potential include gender [25, 26], age [27] and whether the client is considered ‘marginalized’ or ‘integrated’ (e.g. [28]). There are also likely to be complex interactions between residual barriers such as trauma and other psychological health problems (as recognized by White and Cloud [13] but we do not have sufficient data in the current paper to test these challenges to recovery growth).

Best et al. [15] developed a Recovery Capital measurement model (REC-CAP). The overarching purpose of the REC-CAP is to measure barriers and unmet needs, as well as resources that the individual can use in recovery. To measure levels of recovery capital, REC-CAP combines the Assessment of Recovery Capital (ARC) [18], the Recovery Group Participation Scale (RGPS) [29], the Commitment to Sobriety Scale [30] and the Social Support Scale [31]. The REC-CAP measure has been shown to be a reliable tool for measuring commitment to the cessation of alcohol and drug misuse and should have the capacity to predict future resource needs to meet goals and build long-term recovery motivation. Best and Hennessy [32] have noted that although there has been some progress in the 20 years since the term was first used, there remain fundamental questions about utility and predictive validity as well as around the comprehensiveness of the measures used to capture the concept.

Recovery capital is believed to possess the ability to predict future needs in terms of resources to increase motivation. Lynch et al. [33] have shown that, in a cohort of opiate use disorder patients accessing treatment, there are significant improvements in recovery capital as measured on the ARC, with means scores rising from 37 at enrolment to 43 around three months later (the scale ranges from 0-50). However, change measures have not previously been reported for recovery housing residents, and this may be important to not only predict retention but also support recovery care planning and activities to support completion and the transition back to the community.

Recovery residences provide residential care for people in recovery and help to build recovery capital, and were identified in a review by Humphreys and Lembke [3] as one of three clearly evidenced areas of recovery intervention (along with peer-based recovery support and 12-step mutual aid). These residences focus on the broader aspects of reintegration and community engagement, such as employment and living situations ([34], p. 52), while also addressing the needs of a marginalized client group (see e.g. [28]). The term recovery residences cover a variety of housing models. The National Association of Recovery Residences (NARR) has outlined four levels of recovery residence, based on how they are administered and the level of staffing. Common to all levels is a commitment to abstinence and recovery support, and the provision of communal living arrangements [34].

The current study focuses on Fellowship Living Facilities, which provides recovery residences that are categorized as level 2, or Sober Living Houses (SLH), as an early partner in assessing the implementation of recovery capital measurement in the context of recovery residences, through a partnership with the Florida Association of Recovery Residences, with Fellowship Living a key early adopter in this initiative. Approximately 85% of residents who voluntarily enroll at Fellowship are classified for insurance purposes as ‘indigent’ or otherwise homeless. Depending on the date range, more than 50% were recently released from jail or prison. Many are in need of Speciality Addiction Treatment, but have no health insurance or other financial resources to secure a bed in a residential care facility.

While residing in recovery residences managed by Fellowship Living Facilities, all residents are obliged to attend regular 12-Step meetings. According to the house guidelines, the resident is also required to actively seek employment, and to secure a job within two weeks. Fellowship Living Facilities has a strict zero tolerance of drug and alcohol use, and only sober visitors are allowed to visit the residents. Longitudinal studies on recovery residences [35, 36] and Sober Living Houses [37–39] have shown that increased rates of employment and lower levels of involvement by criminal justice entities are associated with long-term stays in the residences, but that the social dynamics and networks of residents might influence the amount of time retained [40, 41].

As well as examining recovery capital change in recovery residence settings, there is a need for further research into changes in recovery capital, based on sub-group characteristics and on support engagement factors. This paper addresses three research questions:

1. Which recovery capital factors near the time of admission are associated with retention in the recovery residences?
2. How does recovery capital change during the first six months of residence?
3. What demographic and behavioral characteristics are associated with changes in social and personal recovery capital?

## Materials and methods

### Measures

The paper is based on an implementation project using the REC-CAP to assess and monitor changes in recovery capital among clients of recovery residences (with the longer-term goal of using such data to support recovery capital growth. As such, data were collected by house managers in the residence and there was no comparison group and no capacity for following up participants who dropped out of the study. The analysis is based on longitudinal data collected using the REC-CAP, which was self-completed by participants with support from the house managers in the recovery residences where this was needed (for instance, as a result of literacy issues). REC-CAP integrates recovery measures into a self-completion interview schedule. The contextual questions examine demographics (age, sex and ethnicity) and barriers to recovery (using categorical data options). The barriers involved are housing need, historic and current substance misuse, risk-taking, involvement with the criminal justice system, engagement in meaningful activities (such as volunteering or employment) and unmet support and treatment needs (around drug and alcohol treatment, mental health, primary care, family support, and other specialist needs). Likert scale measures (0–20) are incorporated to measure psychological and physical health, quality of life, support network and satisfaction with accommodation, creating a total wellbeing score from 0-100. As noted above, the schedule includes the ARC, which is broken down into personal and social recovery capital, consists of 50 items and has acceptable reported psychometric properties [18]. There are five sub-scales for personal recovery capital (each of five items in the areas of recovery experience; physical health; psychological health; risk-taking and coping) and for social recovery capital (the five sub-scales here are meaningful activities; housing; social support; community involvement and substance use and sobriety).

The purpose of the Recovery Group Participation Scale (RGPS) is to measure one aspect of community capital related to involvement with the recovery community. It contains 14 items that address participation and involvement in community groups, as well as attitude to recovery status [29]. To further account for the importance of social identity and group membership, REC-CAP includes the four-item Social Support Scale [31], while the Commitment to Sobriety Scale [30] is a measure of motivation that also has established psychometric properties. Use of REC-CAP as a research tool involves the completion of 94 individual variables. It takes 15–20 minutes to complete.

***Setting*** Fellowship Living Facilities is a non-profit organization that provides supportive recovery services (with a commitment to a 12-step approach) and Level 2 recovery residences (https://www.fellowshiprco.org) and its sober living facilities are also linked to recovery support services. It consists of a total of 45 houses (seven for women and 38 for men). This equates to 28 beds for women and 152 beds for men. To support recovery, Fellowship Living employs 12 recovery coaches to supplement the primary model which is of mutual, peer support between residents and mentoring from the house manager, who will typically be a more senior peer.

Weekly fees at the time of writing were $185 but this only applied to those who were able to secure employment. Fellowship Living is regulated through the Florida Association of Recovery Residences (NARR) which is a part of the National Association of Recovery Residences (NARR), which requires participation in a formal accreditation programme.

There is no upper time limit to how long a resident can remain with Fellowship Living. However, there are no facilities for parents to have children living with them, and intimate partners are not allowed to stay overnight at the residence. Residents are required to attend mutual aid meetings and to have a 12-step sponsor as pre-requisites for living in the residences. Fellowship Living routinely collects REC-CAP to address recovery progress among the people living in recovery residences.

### Sample

The sample consists of individuals enrolled in a recovery house post-treatment or post-prison, and some clients who continued to attend intensive out-patient treatment in Florida, USA, in the period 2016–2019. All the recovery houses included were managed by Fellowship Living Facilities and offered 12-Step approaches exclusively, with or without the addition of Medication-Assisted Treatment (MAT). The Recovery Coach responsible for each recovery housing unit was responsible for supporting the participant to complete the assessment if needed. All recovery coaches were provided with a full-day training in the model of recovery capital and the REC-CAP tool although their role was only to guide completion of the REC-CAP and to support clients with completion (ie the REC-CAP was not used to support ongoing recovery care planning). The clients completed the online form either with support from the recovery coach in each house or as self-complete. Data were available for 823 individuals who participated in a baseline assessment around the time of admission to the residence. At baseline assessment, 40% were assessed the day they arrived, around 55% were assessed in the same week and around 70% were assessed within a month of arrival at the residence. Around 98% were assessed at some point during the first three months of their stay in the residence. There was no capacity for following up subjects, so only those who remained in the residences at the time of the follow-up interviews completed the REC-CAP forms.

Discharge data for the time period show that the most common reasons for discharge were: “abandoned” (20%), “completed program” (21.3%) and “recurrence of use (relapse)” (18.9 %). Less common reasons for discharge were; “change in network” (0.1%), criminal justice discharge (0.6%), deceased (0.4%), medical discharge (1%), other involuntary discharge (11.5%), other voluntary discharge (12.5%) and referred out (6.5%). Table 1 shows the retention rate after each assessment, with full retention information in the study provided to give the reader some sense of typical durations of stay, although only baseline to 6 months are included in the analysis as a result of attrition. The follow-up assessment (T2) occurred 180 days after the baseline assessment and subsequent assessments occurred at quarterly intervals. Table 2 provides an outline of client recovery capital and demographics at the baseline for the total sample (*n*=823), the retained group (*n*=267) and the group that left the residence before the follow-up assessment (*n*=556). This shows that about one- third (32%) of those originally assessed at baseline were available for a follow-up assessment 180 days later. Thereafter, the number of people assessed nearly halves (ranging from 47-48%) from the second (T2) to the fifth assessment (T5).

**Table 1:** Retention rate at each assessment (N, % of previous assessment sample, % of baseline assessment sample)

Time (Days)	N	% of previous assessment	% of baseline assessment
**Baseline**	823	100	100
**T2 (180)**	267	32	32
**T3 (270)**	129	48	15
**T4 (360)**	62	48	7
**T5 (450)**	29	47	3
**T6 (540)**	10	34	1
**T7 (630)**	2	20	0

**Table 2:** Crude Description of Client Recovery Capital at baseline. Recovery capital characteristics in the retained group (*n*=267) and the group who left the residences before the second assessment (*n*=556)

	Baseline (*n*=823)	Retained group (*n*=267)	Left before second assessment (*n*=556)
	*Mean (SD)/Percent*	*Mean (SD)/Percent*	*Mean (SD)/Percent*
Age	36.81 (11.0)	37.79 (10.3)	36.32 (11.3)
Sex (male)	87.2%	95.1%	83.5% %
Physical health	15.43 (4.7)	15.59 (4.6)	15.39 (4.8)
Psychological health	15.87 (4.6)	16.12 (4.2)	15.74 (4.9)
Quality of Life	14.52 (5.1)	14.87 (4.6)	14.36 (5.3)
Support Network	16.09 (5.1)	16.13 (4.8)	16.07 (5.2)
Accommodation satisfaction	16.29 (4.5)	16.51 (4.1)	16.19 (4.7)
Social Support Scale	23.21 (5.5)	23.48 (5.0)	23.09 (5.7)
Commitment to Sobriety Scale	28.66 (4.1)	28.88 (3.7)	28.55 (4.3)
**Recovery capital**			
Social ARC scale	19.51 (5.8)	20.36 (5.4)	19.80 (6.1)
Personal ARC scale	20.51 (5.5)	20.72 (5.1)	20.41 (5.7)
Total ARC score	40.49 (10.8)	41.07 (9.9)	40.21 (11.2)
RGPS	9.37 (4.5)	10.06 (3.8)	9.04 (4.8)*

*significant at *p*<0.05

### Analytical framework

An initial analysis of the total dataset suggested that variables were incomplete across the two time periods. There were 710 records (86%) with at least one missing value. Housing need was particularly incomplete (54.5% missing data) and was dropped from the dataset. The main area for missing data reflected socio-demographic composition (housing) and drug using behaviours. These reflected issues in data collection and have been subsequently rectified by the treatment providers. The literature suggests that it is optimal to model a complete set of cases [42], so a Multivariate Imputation by Chained Equations (MICE) approach was deployed [43] using a logistic model for binary variables and predictive mean matching modeling to impute continuous variables. The revised analysis was performed on a reduced set of 63 variables (minus the missing block of 27 mainly socio-demographic measures such as housing and drug-using behaviours). The range of variables reflected the REC-CAP dataset that included dichotomous variables (yes/no), interval-level measures such as age and scales encompassing the range of validated measures contained within the schedule. The appropriateness of the imputation models formulated were visually assessed using diagnostic plots.

To examine factors associated with retention, a logistic regression model was conducted on the final imputed dataset using a dichotomized dependent variable for whether the client stayed in the residence for the full six months. The third aim was to explore which variables were associated with changes in social and personal recovery capital using the composite total ARC score. Records that measure the same person at different times are very often correlated, and measures taken closer in time more highly correlated than those taken further apart. Linear mixed models are used with repeated measures data to accommodate both the effect of time and the covariation between records on the same subject at different times [44, 45]. To present standardized effects, the analysis involving the three numerical measures (age, days living in a recovery house, RGPS scale) were divided by their respective standard deviations. The latter were derived using Rubin’s combination rules, appropriate for an imputed dataset [46]. The binary measures are shown as raw coefficients. The analyses were undertaken using Stata v15 [47].

### Ethics

All the participants signed full consent forms. Consent to participate included the client’s permission to use the data for research purposes, along with information that ensured that the data files would be de-identified if ever used for such purposes. The ethical considerations were drafted in adherence with the WMA Declaration of Helsinki [48]. Parts of the data management were conducted at the University of Stockholm in Sweden in a procedure approved by the Swedish Ethical Review Authority (No. 2020-00802).

## Results

### Predictors of retention in the recovery residence

Table 3 shows the logistic regression model conducted to examine the factors associated with treatment retention derived from the multiple imputation model. The model identified five statistically significant factors based on three numerical and two dichotomous measures. For the dichotomous variables, the strongest effect was noted by sex such that the odds of disengaging for females were 3.76 higher than males (95% CI 2.04, 6.90). Clients who sought help with their housing needs were 1.52 times more likely to leave treatment than subjects who did not, with a 95% CI (1.01, 2.29). For numeric variables, there was a significant but weaker effect for age (Odd Ratio [OR] 0.98, 95% CI 0.97, 1.00) and for participation in recovery groups (OR 0.96, 95% CI 0.93-0.99)

**Table 3:** Logistic regression modelling treatment retention (dependent variable = those leaving treatment) derived from the Multiple Imputed Dataset (significant at *p*<0.05)

ARC Factor	Odds Ratio (95% CI)
Age	0.98 (0.97 – 1.00)
Recovery Group Participation Scale	0.96 (0.93 – 0.99)
Seeking more help with housing	1.52 (1.01 – 2.29)
Sex (Female)	3.76 (2.04 – 6.90)

### Changes in ARC scores over time

Initially, paired-sample t-tests were conducted to examine the change in recovery capital between the baseline assessment and the follow-up assessment (T2) for the retained sample. The paired-samples t-tests showed statistically significant growth in all the measured recovery capital scores between the baseline assessment and the first follow-up assessment apart from the social capital ARC score (*p*=0.058) and commitment to sobriety CSS (*p*=0.887). There was a significant (*p*=0.001) average increase in social support and availability of social resources between the baseline (Mean [M]=23.48, Standard Deviation [SD]=5.0) and follow-up assessment (M=24.57, SD=4.9). Furthermore, personal recovery capital showed a significant (*p*=0.006) difference between baseline (M=20.72, SD=5.0) and follow-up (M=21.78, SD=5.0). The total ARC score was calculated by adding clients’ social ARC score and personal ARC score to derive a single scale. This showed a significant (*p*=0.012) increase from baseline (M=41.07, SD=9.9) to follow-up (M=42.91, SD=10.1). RGPS scores were statistically significantly (*P*<0.001) and increased between the baseline assessment (M=10.06, SD=3.8) and the follow-up assessment (M=11.11, SD=3.6).

### Factors associated with changes in recovery capital

Table 4 outlines the linear mixed model conducted to examine factors significantly associated with changes in social and personal recovery capital between the baseline and the follow-up assessment, as operationalized by total ARC score. From the table of regression coefficients (see Table 4), prognostics with a *p*-value lower than 0.05 were selected and refitted to an identical database with the 766 cases augmented by multiple imputation. In the resulting regression, with all 267 subjects included, six statistically significant variables were found to be significantly associated with a change in total ARC score between the baseline assessment and the follow-up assessment.

**Table 4:** Changes in Assessment of Recovery Capital from baseline to 6-month follow-up (significant at *p*<0.05)

ARC Factor	Estimated Coefficients/Effect Estimate (95% CI)
Age	-1.08 (-1.69 - -0.48)
Quality of Life	1.28 (0.62 – 1.94)
Social Support Scale	1.00 (0.20 – 1.81)
Recovery Group Participation Scale	5.40 (4.80 – 6.00)
Full-Time work	1.66 (0.32 – 3.00)
Seeking more Family Support	-3.61 (-5.79 - -1.44)

Furthermore, the linear mixed model showed that six variables significantly affected the amount of change in total ARC score between baseline and follow-up assessment. The aspects positively associated with a change in total ARC score were quality of life, working full time, Social Support and recovery group participation. Age was negatively associated with a change in total ARC score, ultimately indicating that increases in recovery capital were less likely with increased age. Finally, seeking more help for family relationships was also negatively associated with a change in total ARC score, meaning that those who did not seek help for family relationships were likely to increase their level of recovery capital more extensively than those who sought help, suggesting that family issues may reduce recovery capital growth.

To provide more detail to these results, the strongest effect was noted for involvement in recovery groups. The higher the RGPS scale score, the higher the total ARC score. An increase of one standard deviation in the RGPS scale is associated with an increase of 5.4 points in the total ARC score. A strong negative effect was noted for clients seeking extra help from Family Relationships Services such that residents reported on average a total ARC score of 3.61 points lower (95% CI −5.79, −1.44), than people who are not seeking more help. Clients who worked full-time reported an average 1.66 points higher ARC score compared to those who did not, with a 95% CI (0.32, 3.00). Quality of life (QoL) was also found to be significantly associated with a change in total ARC score such that the higher the QoL score, the higher the ARC score. An increase in one standard deviation in Quality of Life is associated with an increase in 1.28 of the total ARC score. In addition, the older a subject is, the lower the total ARC score. An increase in one standard deviation in age is associated with a decrease in 1.08 in the total ARC. The weakest effect was noted for the Social Support Scale where the higher the score, the higher the total ARC score. An increase in one standard deviation in the Social Support Scale is associated with an increase of 1 point in the total ARC score. Overall, a model-based on total ARC means predicted score calculated at the average value of the six selected prognostics is shown below in Table 5. This shows at baseline assessment an ARC score of 42.9 rising to 44.9 at seventh assessment. An F-test for overall equality of the true mean total ARC score across all seven assessment time points was shown to be not statistically significant (F=0.46, *p*=0.84).

**Table 5:** Model-based predicted means for total ARC score

Assessment Number	Mean ARC Score (95% CI)
**1**	42.9 (42.0 – 43.9)
**2**	42.2 (41.3 – 43.1)
**3**	42.2 (40.9 – 43.5)
**4**	42.1 (40.2 – 44.0)
**5**	41.1 (38.3 – 43.8)
**6**	42.8 (38.1 – 47.4)
**7**	44.9 (34.6 – 55.3)

## Discussion

The aim of this study was to assess factors associated with retention in recovery residents and to explore changes in recovery capital among those that were retained.

The logistic regression model suggests that individuals retained in recovery homes are significantly more likely to be older and male, require help with housing and has had greater involvement in recovery groups. The strongest effect on disengagement was noted for females who were nearly four times more likely to disengage compared to males. There is considerable literature highlighting the severity of comorbidities faced by women in substance use services relating to mental health issues, and physical or sexual abuse [49–51] which has been shown to adversely affect engagement levels [52]. Cloud and Granfield [12] suggested that psychological and social aspects of the female experience of recovery from substance misuse differ from the male experience. Neale et al. [25] studied gender-specific expressions of recovery capital and found both differences and similarities between male and female recovery capital pathways. In their study of social recovery capital among homeless drug and alcohol users, Neale and Stevenson [26] found that women have a closer relationship with their families compared to men and a larger social network. The current findings and existing research demonstrate the importance of further exploration of gender-specific recovery pathways and the impact of early female departures from recovery residences on women’s recovery trajectories [53, 54].

A strong effect on engagement was also noted in relation to housing needs such that people identified with accommodation issues were nearly twice as likely to disengage relative to those with no needs. The relationship between housing and recovery has been well documented [55, 56]. The nature of participants’ housing needs, possibly related to families, will be the subject of further research by placing housing satisfaction as the basis for building ongoing and sustained recovery. Enhanced, case-managed pathways to support people with housing needs should be integrated within the range of options offered.

The longitudinal comparisons showed non-significant increases in recovery capital with enablers identified among the individuals retained sample in this study, of increased recovery capital associated with recovery group participation, working full time, a higher quality of life and greater social support. These findings build on previous research which has reported the benefits of recovery residences in building the resources required to build and sustain a recovery journey [35, 37, 41], by reporting improvements in recovery capital scores for those retained in Fellowship Living to the follow-up assessment point. However, the findings add to the currently available literature on recovery capital by demonstrating predictors of recovery capital change addressing one of the issues raised in Hennessy [20]. One exception to this was commitment to sobriety, which remained at the same level between the baseline and the follow-up assessment, although this may be the result of a ceiling effect as entering, as well as remaining, in the residence requires an established commitment to sobriety.

Changes in recovery capital also show that, among the residents who remained, the strongest enabler is amongst those that participate in recovery groups. Living in recovery residences is about active participation in a recovery community and often requires mutual aid engagement, and “residents’ bond as a community and support one another’s recovery” [57]. The findings are consistent with existing findings on the importance of developing a support network that is conducive to recovery [15] and of actively participating in and belonging to a recovery group [58, 59]. Studies of recovery progress in recovery residences suggest that a longer stay is associated with improvements in areas such as substance use, employment and self-discipline [60], as well as improved monthly income and lower incarceration rates [35].

The current study builds on these findings by demonstrating significant progress in building recovery capital for people who remain in recovery residences facilitated by engagement in mutual aid recovery groups. Furthermore, research on social capital in recovery residences has primarily focused on social networks and relationships within the residence (e.g. [40, 41]). However, a strong barrier that reduced recovery capital was noted in the perceived need for more family support. The findings in the present paper suggest that relationships outside the residence, such as stable family relationships, are also important for improvements in recovery capital including greater involvement in the family as an active participant in the recovery process [61]. As the greater need for help in the area of family support is significantly correlated with lower ARC scores, one potential area for focus for recovery residence coaches could be in supporting positive family relationships and providing pathways to family support services where these are needed. However, as this was not the focus of the study, we do not know whether seeking help for family issues should be regarded as a positive and this issue requires further investigation. Furthermore, it is likely that seeking help for family relationships is an expression of the established notion that social capital, such as family relationships, is an essential resource for recovery progress [12]. Ongoing involvement in family support services could indicate relationship problems that are acting as a barrier to effective recovery and reintegration pathways. It is also likely that ongoing family problems may be a catalyst for early drop-out from recovery residences if the individual feels the need to actively re-engage with the family. The findings on changes in recovery capital also show a relatively strong positive effect for participants in full-time employment. For people in recovery, engagement in work has been shown to be affected by a range of comorbid chronic conditions [62] creating barriers to stable employment [63]. The need for integrating employment support within recovery residences should be further encouraged.

Age can be shown to be a consistent factor that is associated with disengagement and with improvements in recovery capital. The current findings extend the existing evidence base by demonstrating that recovery progress is likely to vary based on a range of factors including sex and age. These are interesting findings from a practice and policy perspective. Older age has previously been considered ‘negative recovery capital’ [12], but this only partly fits with the results. While older males are more likely to be retained, they are less likely to show the same levels of recovery capital growth as younger residents, indicating that the older age group has more recovery barriers to overcome. For example, men are less likely than women to have a close relationship with their families [25, 26]. A different service focus therefore might be required for the older males, who are seemingly more likely to stay but not to progress to the same extent as younger people and women. There is considerable scope for the development of targeted interventions to be delivered by house managers in recovery residences to support the age-specific needs of residents and for the managers of women’s houses to be trained in motivational approaches including group engagement to support retention in this population.

### Limitations and future research

Longitudinal data were only available for the individuals who continued to live in the residential service, which means that the analysis of outcomes is only available for those who remained although this does allow for an exploration of factors associated with engagement. This is a particular problem given the high attrition rate, which we are assuming as a negative but that will remain an assumption until we have outcome data beyond the point of discharge from the residences, and can test the association between duration of retention, reason for discharge and outcomes. This is a particular issue around ethnicity and resulting issues of intersectionality and this is something we would hope to examine as our work in this area develops.

Large surveys often suffer from a degree of non-completion and this one was no exception. To address this, a statistical method (MICE) was applied to impute missing values. The survey has been enhanced as a result as it was possible to use all the cases. Some key prognostics were not imputed however including a key measure related to housing and some drug-using indicators which will limit the explanatory power for the analysis. There was also considerable variation in time to baseline data collection with around one-third of participants not completing the baseline survey until after one month of living in the residence. This may have influenced the extent to which the summary data represent a true baseline and may have impacted on the change analysis.

In addition, as enhanced data comes on line future work could consider use of hazard models to supplement the analysis of engagement. We are also assuming that growth in recovery capital is a consequence of living in recovery residences and following the program it recommends, but we have to be aware of the potential benefits of completing recovery capital assessments and future research should address this potential artefactual effect. The current REC-CAP also does not collect data on socio-economic status and this may limit analysis around population sub-groups and is something that will be addressed in future versions of the scale.

It is important to note that all the data were collected from Sober Living Houses managed by Fellowship Living Facilities. Sober Living Houses are a specific type of recovery residence and it is likely that different profiles of change would be found in other settings, and in this case from a single provider of Sober Living facilities. Furthermore, retention in the residences has been interpreted as a positive recovery outcome even though the recovery progress among those who left the residences is unknown. The reasons for discharge are illustrative of a heterogeneous group leaving treatment, and include both relapse and completion of the program. This further implies that the group’s members who were not assessed comprise individuals with a variety of recovery trajectories. Future research will need to transition to an Intention To Treat model (where participants are followed up regardless of their treatment status and based on ongoing participation in the research study regardless of their duration of treatment engagement) to address the impact of retention on recovery capital more effectively.

Previous research suggests that the implications, experience and availability of recovery capital vary by sex (see for example [25]). The sample in the present paper has certain limitations when comparing men and women, as it is based on a relatively small number of women retained to follow-up. To build on existing, mainly qualitatively based, knowledge, future research should aim to address the suggested gender-based differences, as well as their implications for the treatment and service environment, and the impact of additional initiatives addressed specifically at the retention of women and young people.

Finally, the scientific development of recovery capital in practical environments, in terms of quantifications and assessment tools, is still at an early stage. Researchers have found quantifications of recovery capital, such as through the REC-CAP, analytically valuable for analyzing recovery progress and change. The next step will be to examine further its potential contribution to practical treatment and recovery support environments, and in particular how clients and practitioners can benefit from its implementation. At present, we cannot state with any confidence what the implications of score changes are but we are looking to do prospective outcome studies that will attempt to address the impact of recovery capital score changes on a range of recovery outcomes.

## Data Availability

Data can be made available on request and is housed at the Recovery Outcomes Institute.
